# Energy-Mix Entropy and the Scale–Composition Divide in Urban Renewable Transitions: Evidence from Chinese Cities

**DOI:** 10.3390/e28090971

**Published:** 2026-09-01

**Authors:** Niwei Ma, Qing Mo, Fenglie Xi, Ying Liang, Yuan Li

**Affiliations:** College of Tourism & Urban Rural Planning, Xichang University, Xichang 615000, China

**Keywords:** renewable energy transition, Shannon entropy, energy-mix diversity, urban capabilities, digital infrastructure, green innovation, distributional heterogeneity, Chinese cities

## Abstract

Cities can consume more renewable energy without achieving an equivalent shift in their energy mix. We examine this scale–composition divide in a balanced panel of 284 Chinese cities from 2007 to 2019 and use normalized Shannon entropy to characterize the state of each city’s energy portfolio. The analysis distinguishes absolute renewable consumption, total renewable share, and wind plus solar share; it also separates digital infrastructure, digital financial inclusion, and environmental digital governance. Total energy use is strongly positively associated with absolute renewable consumption, negatively associated with total renewable share, and positively associated with wind plus solar share. Energy-mix entropy has a nonlinear association with renewable composition that persists across lagged and alternative diversity measures. By contrast, upper–lower quantile differences, formal subgroup contrasts, and capability interactions based on entropy excluding renewable carriers do not survive multiple testing adjustment. The digital measures also diverge in a common sample: digital financial inclusion is negative, environmental digital governance is positive, and digital infrastructure is imprecisely estimated. Urban renewable transition is therefore better understood as a compositional process associated with portfolio state than as the uniform accumulation of city capabilities.

## 1. Introduction

A city can use more renewable energy without moving decisively away from incumbent fuels. This distinction matters in China, where national low carbon objectives are implemented through local economies that differ sharply in industrial structure, infrastructure, and resource endowment [1,2,3,4]. Growth in renewable volume may reflect a larger energy system; a compositional transition requires renewables to gain relative weight within that system. The empirical puzzle is therefore not simply why some cities consume more renewable energy, but why renewable expansion translates into energy-mix change in some urban systems and not in others.

Two measurement choices have obscured this puzzle. First, absolute renewable consumption conflates scale with substitution. A large city may consume more renewable energy than a smaller city while retaining a lower renewable share. Hydropower also follows a different geography, infrastructure, and policy history from wind and solar power, so a pooled renewable total can conceal technologically meaningful variation. Second, studies often group broadband access, digital finance, and public sector digital capacity under broad labels such as digitalization or digital governance. Yet broadband subscriptions measure infrastructure access, digital financial inclusion measures access to and use of financial services, and environment-related digital procurement captures a narrower form of administrative capacity [5,6,7,8,9,10]. Conflating these constructs can turn distinct mechanisms into an apparently coherent but misleading narrative.

The paper addresses these measurement problems through a testable complex systems argument. Calling cities complex adaptive systems adds little unless system state is observed and the implied relationships can be rejected [11]. We characterize system state by the normalized Shannon entropy of a city’s final energy mix [12,13]. The index measures how evenly final energy use is distributed across carriers: concentration produces low entropy, whereas diversification produces high entropy. It is not a normative measure of disorder or governance quality. The central question is whether renewable composition varies nonlinearly with this portfolio state and whether capability associations depend on it.

This framing replaces a universal capability hypothesis with competing mechanisms. Finance may fund renewable investment, but it may also sustain incumbent production, real estate, and consumption growth [14,15]. Digital finance can ease financing frictions while expanding energy-intensive consumption [16,17,18]. Human capital may support technological absorption, yet broad higher education enrollment need not measure the skills required to install, operate, and coordinate local energy technologies [19,20,21]. Green patents indicate knowledge production, but deployment still depends on complementary infrastructure and institutions [22,23,24]. The empirical signs are therefore informative precisely because theory does not predetermine that every capability must promote renewable composition.

We study 284 Chinese cities from 2007 to 2019. Renewable final energy share is the principal outcome; absolute renewable consumption and wind plus solar share distinguish scale from alternative forms of composition. The capability measures cover digital infrastructure, industrial advancement, financial development, income, human capital, green innovation, digital financial inclusion, and environmental digital governance. City and year fixed effects absorb permanent city attributes and common shocks, while registered population and four policy pilot indicators capture observable changes in scale and policy exposure.

The central contribution is to show that urban renewable expansion and compositional transition are empirically distinct and that the latter is nonlinearly associated with energy portfolio state. Three design choices make that claim interpretable. We compare absolute renewable use, total renewable share, and wind plus solar share; we keep three digital constructs separate and compare them on an identical 2015–2018 sample; and we operationalize system state with Shannon entropy, testing the result with a lag, Simpson diversity, and entropy calculated after removing renewable carriers. Quantile and subgroup analyses then serve as boundary tests: city-clustered bootstrap, false discovery rate adjustment, and direct coefficient difference tests determine whether visually heterogeneous patterns support formal claims.

The evidence narrows the cumulative capability account. Total energy use predicts more renewable consumption in absolute terms but a lower total renewable share and a higher wind plus solar share. Energy-mix diversity has a robust nonlinear association with renewable composition, supporting H1. Digital infrastructure is positive in the joint fixed-effects model, green innovation and environmental digital governance are positive in their available samples, and financial development and digital financial inclusion are negative in the main share models; most first-difference estimates are null. Moreover, adjusted upper–lower quantile contrasts, direct subgroup tests, and entropy interactions that exclude renewable carriers do not support general distributional, structural, or complementarity claims. The durable finding is system state nonlinearity, not universal capability accumulation.

Section 2 develops the system state argument and its competing mechanisms. Section 3 links each empirical step to a distinct question concerning average associations, timing, composition, nonlinearity, distribution, and structural context. Section 4 presents the evidence in that sequence. Section 5 interprets the supported and unsupported propositions and derives state-contingent policy implications. Section 6 concludes.

## 2. Theoretical Framework and Hypotheses

### 2.1. Urban Transitions as State Dependent Energy Systems

Urban energy transitions unfold within systems whose components adjust at different speeds. Energy networks and industrial equipment are durable, digital access and finance can expand quickly, and human capital and institutional learning accumulate over longer horizons. These temporal differences create path dependence: the same nominal capability may be consequential in one portfolio configuration and largely inert in another. An average coefficient can summarize the sample, but it cannot by itself reveal whether the underlying relationship changes with system state.

We represent system state along two related dimensions. Renewable share records the position of hydro, wind, and solar within final energy use, while normalized Shannon entropy records the concentration or dispersion of the full ten carrier portfolio. Introducing a new carrier into a concentrated system may require substantial infrastructure and coordination; a diversified system may already possess interfaces, market rules, and operating routines that lower those costs. Diversification can also increase coordination burdens and exposure to energy security risks [25]. Because these forces work in opposing directions, nonlinearity is a more discriminating prediction than a uniformly positive entropy effect.

Composition also reflects inherited geography and infrastructure. Hydropower-rich cities can occupy the upper tail of total renewable share because of resource endowment, whereas expanding wind and solar represents a different transition path. China’s district heating systems illustrate how durable infrastructure can lock cities into particular fuels and how diversification may relax some forms of carbon lock-in [26]. City-level studies likewise document persistent transition differences and uneven local policy readiness [27,28]. These considerations motivate separate measures of total renewable share and non-hydropower renewable share.

**H1 (**system state nonlinearity**).**  *The association between energy-mix diversity and renewable transition composition is nonlinear rather than uniformly monotonic.*

### 2.2. Distinct Capabilities and Competing Mechanisms

Digital infrastructure, digital finance, and environmental digital governance describe different capacities. Broadband access may support load forecasting, distributed resource coordination, and access to digital services, although its net energy implications remain contested [5,7,8,29,30]. Environmental digital governance instead captures public procurement of digital tools for environmental administration [9,10], while digital financial inclusion captures the breadth and depth of digital financial services [16,31]. Their mechanisms differ, so neither theory nor measurement requires their coefficients to share a sign.

Digital finance makes the competing mechanisms especially clear. Easier access to payments and credit can reduce financing frictions and support renewable innovation [16,18]. The same expansion can stimulate household consumption and associated energy demand [17]. A negative association with renewable share can therefore coexist with wider financial access or stronger innovation. It should not be interpreted as a direct measure of governance quality.

Green innovation and human capital capture different parts of technological capability. Green patent applications indicate locally produced knowledge, and patent citation evidence shows that renewable technology spillovers vary across technologies and extend beyond power generation [24]. Higher education enrollment is a broader measure of learning capacity. Evidence on green skills suggests that transition readiness depends on the match between existing tasks and technology-specific requirements [21]. General enrollment may consequently have a weaker or less stable association with renewable deployment than directly observed green innovation.

Financial development, income, and industrial advancement are similarly ambiguous. A deeper financial system may lower capital costs and distribute transition risk [14,15], but it may also reinforce incumbent sectors. Higher income expands fiscal and investment capacity while raising electricity and transport demand. A shift toward services can reduce the relative weight of heavy manufacturing, yet construction and service activity still consume energy. These countervailing channels make the direction and magnitude of each association an empirical question.

**H2 (**distributional heterogeneity**).**  *The conditional association between an urban capability and renewable transition composition varies across the outcome distribution and need not have a common sign at all quantiles.*

### 2.3. Capability System Complementarities

The stronger complexity proposition concerns contingency rather than accumulation. A capability may matter because of the system in which it operates. Broadband infrastructure, for example, may become more useful when diverse energy flows increase the value of measurement and coordination. Finance may support incumbents in a concentrated fossil system but fund investable renewable projects in a more diversified portfolio. Industrial restructuring may facilitate non-hydropower deployment only where networks and operating routines can accommodate new technologies.

We test this proposition in two complementary forms. The first interacts each capability with standardized energy-mix entropy and asks whether its conditional association changes with portfolio diversity. The second summarizes digital infrastructure, industrial advancement, finance, human capital, and green innovation in an equal-weight capability bundle, then estimates its level, squared term, and interaction with entropy. The bundle is a parsimonious stress test of cumulative capability, not a validated latent construct or a claim that its components carry equal policy weight.

**H3 (**capability complexity complementarity**).**  *The association between selected urban capabilities and renewable transition composition depends on the diversity of the local energy system.*

### 2.4. Structural Heterogeneity and Rival Explanations

Observed capability patterns may also reflect structural sorting. Eastern cities tend to have deeper markets and denser innovation networks; resource-based cities face stronger incumbent industry constraints; and core cities differ from peripheral cities in administrative status, labor markets, and infrastructure. Agglomeration, resource endowment, policy exposure, and urban hierarchy can therefore mimic cumulative capability. A high renewable share quantile may likewise reflect hydropower geography rather than superior governance. Subgroup and quantile comparisons are consequently treated as tests against a single upward development narrative, not as city rankings.

**H4 (**structural heterogeneity**).**  *Capability transition associations differ across eastern, central, and western regions; core and peripheral cities; and resource-based cities and non-resource cities.*

**Figure 1:**
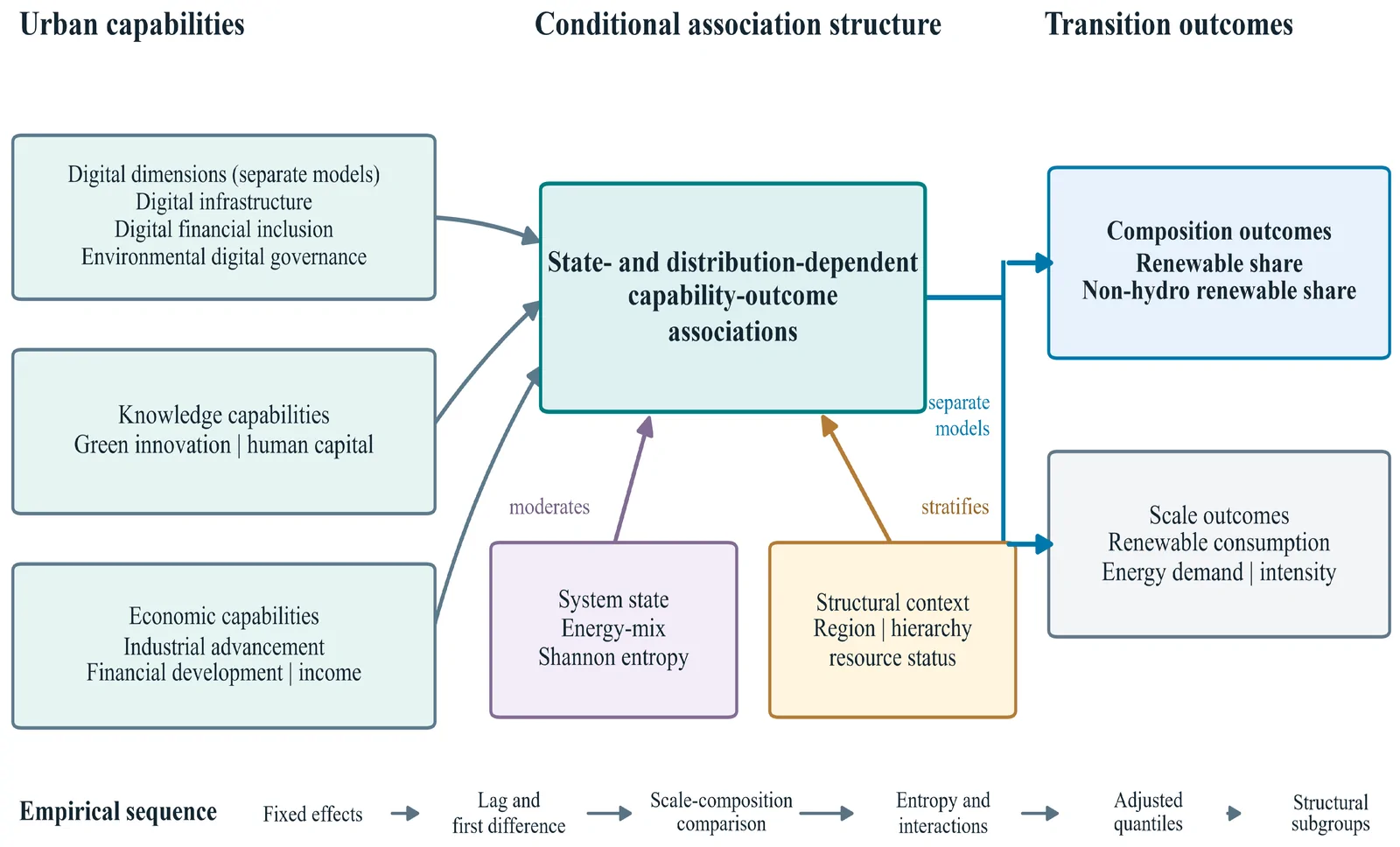
Evidence aligned framework. Urban capabilities are estimated separately; energy-mix entropy represents system state diversity; scale and composition outcomes are distinguished. The bottom row gives the empirical sequence.

The resulting evidence-aligned analytical framework and empirical sequence are summarized in Figure 1.

## 3. Data, Measurement, and Empirical Strategy

### 3.1. Sample and Data Provenance

The unit of observation is a prefecture-level-or-above city-year. The balanced core panel contains 284 Chinese cities observed from 2007 to 2019, yielding 3692 observations. Final energy data come from the comprehensive city dataset developed by Yang et al. [3] and cover ten carriers: coal, oil, natural gas, thermal power, heat, other energy, hydropower, wind power, solar power, and nuclear power. Hydropower, wind, and solar form the renewable components used in the principal measure.

Economic, industrial, demographic, banking, education, and broadband variables come from the public replication workbook associated with Li et al. [32] and are cross-checked against the China City Statistical Yearbook. The repository description lists 287 cities, whereas the downloaded workbook contains 284 unique cities; the observed count defines the balanced panel. Green patents cover 283 cities in 2007–2018, digital financial inclusion covers 284 cities in 2011–2019, and environmental digital governance matches 280 cities in 2015–2019. To distinguish digital constructs from sample composition, their strict common sample comparison uses 1120 observations for 280 cities in 2015–2018. Table 1 reports the variable definitions and coverage, while Supplementary Table S8 documents the sample flow. No series is extended beyond its source coverage.

The baseline controls include four policy indicators: Low Carbon City Pilot, Broadband China, Big Data, and Information Benefit programs. Each equals one for a designated city from its applicable treatment year onward. City fixed effects absorb time-invariant geography, inherited resource conditions, and other permanent differences; year fixed effects absorb shocks common to all cities. Registered population captures changing city scale. Structural comparisons use the source classifications for resource-based status, core city status, and eastern, central, and western regions.

### 3.2. Renewable Transition Outcomes

The principal outcome isolates composition by expressing renewable final energy consumption as a share of total final energy:

(1) $${RED}_{it}=\frac{{Hydro}_{it}+{Wind}_{it}+{Solar}_{it}}{{EC}_{it}}$$

where ${RED}_{it}$ is the renewable share for city i in year t; ${Hydro}_{it}$, ${Wind}_{it}$, and ${Solar}_{it}$ are final energy consumption from hydropower, wind, and solar, respectively; ${EC}_{it}$ is total final energy consumption across all ten carriers; and subscripts *i* and *t* index cities and years, respectively. This ratio measures composition rather than physical scale. The first alternative outcome excludes hydropower:

(2) $${REDNH}_{it}=\frac{{Wind}_{it}+{Solar}_{it}}{{EC}_{it}}$$

In Equation (2), ${REDNH}_{it}$ is the non-hydropower renewable share, and the letters NH denote non hydropower; ${Wind}_{it}$, ${Solar}_{it}$, ${EC}_{it}$, *i*, and *t* retain the definitions given after Equation (1). The second alternative is ln(1 + Hydro + Wind + Solar), which retains absolute renewable consumption. Comparing these outcomes addresses the concern that larger and richer cities may use more of every energy carrier. Energy intensity is measured as ln(EC/GDP).

### 3.3. Energy Portfolio Entropy

For each city *i* and year *t*, normalized Shannon entropy is:

(3) $$H_{it}=-\frac{\sum_{k=1}^{K}s_{kit}ln\left(s_{kit}\right)}{ln\left(K\right)}$$

where $H_{it}$ is normalized Shannon entropy; *K* is the number of energy carriers in the relevant portfolio; *k* indexes a carrier; $s_{kit}$ is the share of carrier *k* for city *i* in year *t*; ∑ denotes summation from *k* = 1 to *K*; and ln denotes the natural logarithm. For the full energy mix, *K* = 10; for the renewable portfolio comprising hydropower, wind, and solar, *K* = 3. The normalization bounds the index between zero and one. A value near zero indicates concentration in one carrier; a larger value indicates a more even distribution across carriers. These indices measure portfolio diversity, not policy quality or transition performance. Because the shares used to construct entropy are related to the outcome shares, entropy models are interpreted as system state descriptions rather than as causal treatment effects.

Because full-mix entropy and renewable outcomes share components, robustness checks use (i) Shannon entropy recalculated over seven non-renewable carriers after excluding hydropower, wind, and solar, (ii) normalized Simpson diversity for all ten carriers, and (iii) one-year-lagged full-mix Shannon entropy. These indices describe portfolio configuration, not policy quality or a causal treatment.

### 3.4. Urban Capability Measures

The three digital variables are measured and modeled separately. Digital infrastructure is ln(1 + broadband subscriptions per 100 registered residents). Digital financial inclusion is the Peking University index of coverage breadth, use depth, and degree of digitalization [31]. Environmental digital governance is the cumulative value of procurement contracts jointly related to digital technology and environmental governance, divided by GDP [33]. Keeping these variables separate prevents infrastructure access or financial service reach from being interpreted as general governance quality.

Green innovation is ln(1 + green invention and utility model patent applications). Human capital is regular higher education enrollment as a percentage of registered population. Financial development is year-end bank deposits plus loans divided by city GDP; income is logged GDP per capita; and industrial advancement is tertiary industry value added divided by secondary industry value added. Continuous predictors are standardized within each estimation sample, so a coefficient gives the outcome difference associated with a one-standard-deviation difference in the predictor.

### 3.5. Average Associations, Temporal Ordering, and Within-City Change

The empirical sequence begins with the average within-city association:

(4) $$Y_{it}=\beta X_{it}+\gamma^{\prime }C_{it}+\alpha_{i}+\lambda_{t}+\varepsilon_{it}$$

where $Y_{it}$ is the modeled renewable outcome for city *i* in year *t*; $X_{it}$ is one capability variable standardized within its estimation sample; β is the coefficient on $X_{it}$; $C_{it}$ is the applicable control variable column vector, comprising standardized logged registered population, standardized logged GDP per capita unless income is $X_{it}$, and the four binary policy indicators; γ is the corresponding coefficient vector; the prime symbol ′ denotes transposition, so the product is the control vector inner product; $\alpha_{i}$ and $\lambda_{t}$ are city and year fixed effects; $\varepsilon_{it}$ is the disturbance term; and subscripts *i* and *t* index cities and years. Standard errors are clustered by city. A joint core model includes digital infrastructure, industrial advancement, financial development, income, and human capital simultaneously. Green innovation and the two additional digital constructs are estimated in their available samples to avoid discarding earlier years from the core panel.

One-year-lag models replace $X_{it}$ with $X_{i,t-1}$, where subscript *t* − 1 denotes the preceding year. They test whether the predictor temporally precedes the outcome but do not establish exogeneity. First-difference models estimate annual changes with year fixed effects. These models remove time-invariant levels more directly but amplify short-run noise and measurement error. The interpretation is therefore based on the pattern across specifications rather than on one preferred significance threshold.

### 3.6. System State Nonlinearity and Capability Contingency

The second stage tests whether renewable composition varies nonlinearly with energy portfolio state:

(5) $$Y_{it}=\beta_{1}H_{it}+\beta_{2}{H_{it}}^{2}+\gamma^{\prime }C_{it}+\alpha_{i}+\lambda_{t}+\varepsilon_{it}$$

where $H_{it}$ is the selected entropy index standardized within the estimation sample; ${H_{it}}^{2}$ is its squared term; $\beta_{1}$ is the coefficient on the linear entropy term; and $\beta_{2}$ is the coefficient on the squared term. The symbols $Y_{it}$, $C_{it}$, γ, $\alpha_{i}$, $\lambda_{t}$, $\varepsilon_{it}$, *i*, and *t* retain the definitions given after Equation (4). H1 is evaluated from the joint significance and shape of the linear and squared terms. Capability contingency is evaluated with:

(6) $$Y_{it}=\beta X_{it}+\delta H_{it}+\theta \left(X_{it},\times ,H_{it}\right)+\gamma^{\prime }C_{it}+\alpha_{i}+\lambda_{t}+\varepsilon_{it}$$

In Equation (6), $X_{it}$ and $H_{it}$ are the standardized capability and full energy-mix entropy variables; β is the capability coefficient when $H_{it}$ = 0; δ is the entropy coefficient when $X_{it}$ = 0; θ is the coefficient on the capability-by-entropy interaction; and × denotes multiplication. The symbols $Y_{it}$, $C_{it}$, γ, $\alpha_{i}$, $\lambda_{t}$, $\varepsilon_{it}$, *i*, and *t* retain the definitions given after Equation (4). The interaction *p* values are adjusted using the Benjamini–Hochberg false discovery rate procedure across the capability interaction family. The capability bundle model adds the squared-bundle term and bundle-by-entropy term.

The capability bundle is the equal-weight arithmetic mean of the standardized digital infrastructure, industrial advancement, financial development, human capital, and green innovation measures. The interaction family and bundle model are repeated with seven-carrier entropy that excludes hydropower, wind, and solar.

### 3.7. Distributional and Structural Boundary Tests

The distributional analysis asks whether an average association masks systematic differences across the conditional renewable share distribution. The outcome and each predictor are first residualized with respect to the controls and city and year fixed effects. Quantile regressions are then estimated at tau = 0.10, 0.25, 0.50, 0.75, and 0.90; these positions describe conditional outcomes, not permanent city stages. The 95% confidence intervals are obtained from 99 citys clustered bootstrap replications conditional on residualization, while Supplementary Table S7 tests the tau = 0.90 minus tau = 0.10 contrast with false discovery rate adjustment. Local Gaussian estimates centered at predictor quantiles theta = 0.10, 0.25, 0.50, 0.75, and 0.90 use a 0.35-standard-deviation bandwidth and are interpreted as descriptive maps rather than 25 separate hypothesis tests.

The structural analysis asks a different question: whether capability coefficients differ across regions, city hierarchy, or resource status. Subgroup fixed-effects models use one-year-lagged predictors, common full-sample standardization, and the baseline controls. Group specific estimates are reported in the results section. Supplementary Table S5 directly tests coefficient equality with city-clustered variances and adjusts the contrast family for multiple testing. Significance in one subgroup but not another is never treated as evidence of a coefficient difference.

### 3.8. Identification and Interpretation Boundary

This design separates several questions that a single estimator cannot answer. Fixed effects characterize average within-city associations; lags and first differences probe timing; alternative outcomes separate scale from composition; entropy models test system state nonlinearity; and quantile and subgroup models evaluate distributional and structural claims. The controls absorb permanent city attributes, common shocks, population change, and four observed policy pilot exposures. They do not eliminate time-varying confounding, reverse causality, policy endogeneity, migration sorting, or intercity spillovers. Because no prespecified spatial weights matrix is available, spatial dependence is not estimated. All coefficients are therefore interpreted as conditional associations rather than causal effects.

Data processing and statistical analyses were performed using Python (version 3.12.13), with NumPy (version 2.3.5), pandas (version 3.0.1), SciPy (version 1.18.0), statsmodels (version 0.14.6), and scikit-learn (version 1.9.0). Figures were generated using Matplotlib (version 3.11.1).

## 4. Empirical Results

### 4.1. Descriptive Evidence and the Composition Problem

Table 2 summarizes the distributions and sample coverage of the principal study variables.

The outcome distributions reveal the measurement problem before any regression is estimated. The mean total renewable share is 0.0522, with an interquartile range of 0.0151–0.0780, whereas the mean non-hydropower share is only 0.0081. Hydropower therefore accounts for much of the measured renewable position of many cities. Full energy-mix entropy averages 0.6219 and ranges from 0.3682 to 0.8395; renewable-portfolio entropy averages 0.3670 and is more dispersed. A high total renewable share cannot, on these distributions alone, be read as broadly advanced wind and solar deployment.

**Figure 2:**
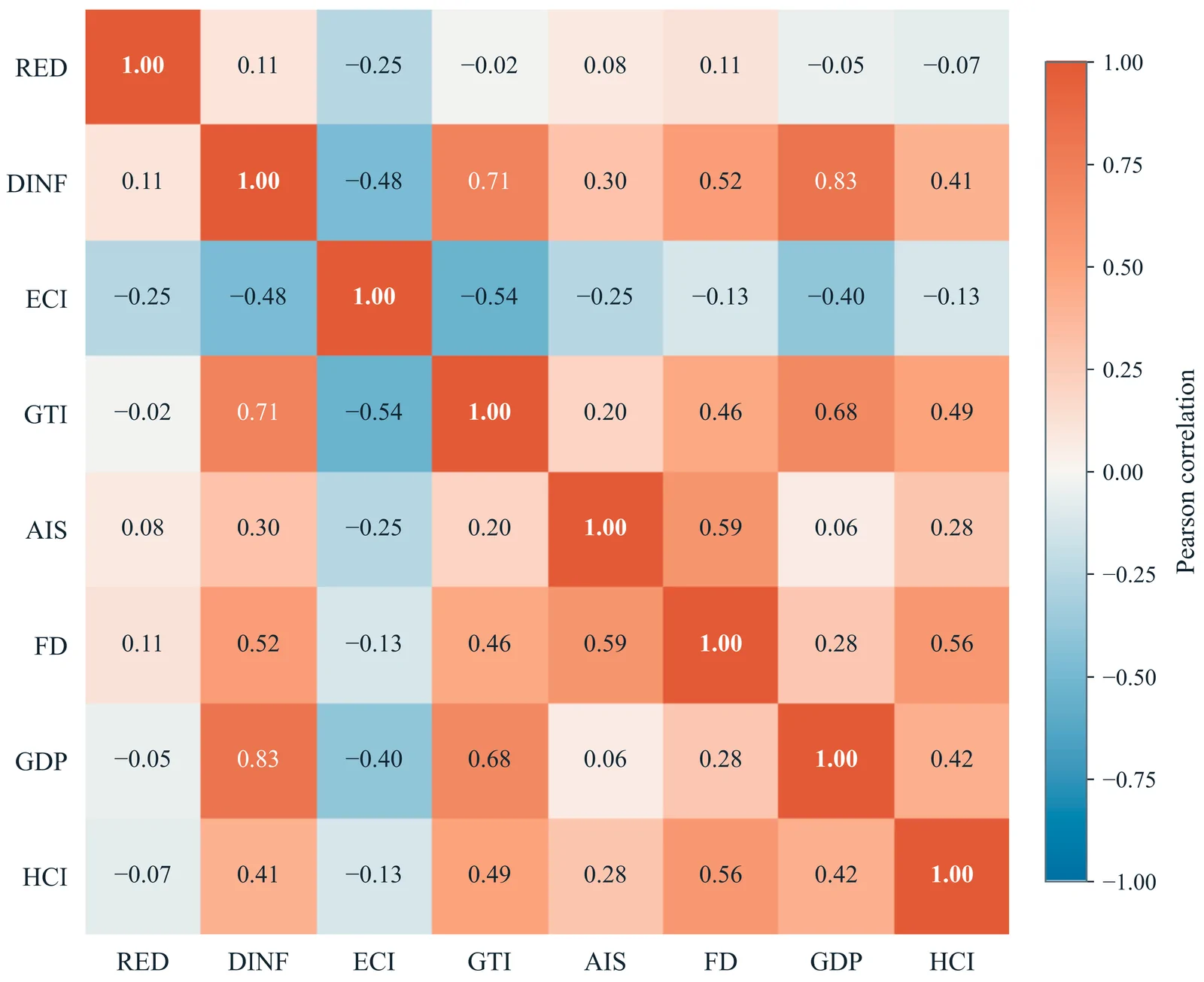
Pairwise correlation structure in the formal city panel. Correlations are descriptive and do not include city or year fixed effects.

Figure 2 presents the pairwise correlation matrix of the principal study variables. Several capabilities cluster in city level data. These pairwise correlations are descriptive and do not absorb permanent city attributes or common shocks. Their main implication is design-related: correlated development indicators should not be interpreted one at a time as independent urban capacities. The fixed-effects models therefore standardize predictors, control for population and policy exposure, and estimate a joint core specification. The scale–composition tests later in this section directly address the additional correlation between economic size and energy use.

### 4.2. Capability Associations in the Average City

Table 3 reports separate and joint city and year fixed-effects estimates for renewable share.

The average within-city estimates already reject a uniformly positive capability account. Green innovation is positively associated with renewable share (beta = 0.00355, *p* = 0.0057), a 0.36 percentage point difference for a one-standard-deviation increase. Environmental digital governance is also positive in its 2015–2019 sample (beta = 0.00192, *p* = 0.0069). Digital infrastructure is positive but imprecisely estimated when entered separately (beta = 0.00335, *p* = 0.110), while industrial advancement and human capital are indistinguishable from zero.

Finance moves in the opposite direction. Financial development is associated with a 0.44 percentage point lower renewable share (*p* = 0.0059), and digital financial inclusion with a 1.43 percentage point lower share in 2011–2019 (*p* = 0.0011). These coefficients do not imply that finance is intrinsically harmful. The measures capture broad banking depth and digital financial access, not the allocation of credit to renewable projects. Their negative signs are consistent with finance supporting incumbent production, construction, or consumption as well as transition investment [14,16,17].

The joint core model confirms that these indicators are not interchangeable. Conditional on the other core capabilities, population, policy exposure, and city and year effects, digital infrastructure is positive (beta = 0.00391, *p* = 0.0375) and financial development remains negative (beta = −0.00485, *p* = 0.0035). Industrial advancement, income, and human capital remain imprecisely estimated. The result is a configuration of countervailing associations, not an additive capability ladder.

### 4.3. Timing and Within-City Changes

Table 4 compares one-year-lagged estimates with first difference estimates to distinguish temporal ordering from within-city changes.

Temporal ordering preserves three of the clearest signs. One-year-lagged green innovation is positive (beta = 0.00328, *p* = 0.0092), digital financial inclusion is negative (beta = −0.01071, *p* = 0.0012), and environmental digital governance is positive (beta = 0.00140, *p* = 0.0360). Lagged digital infrastructure and financial development retain their contemporaneous signs but are estimated less precisely.

Annual changes provide a stricter boundary. Digital financial inclusion remains negative (beta = −0.00085, *p* = 0.0330) and environmental digital governance remains positive (beta = 0.00070, *p* = 0.0026), but the other first-difference estimates are indistinguishable from zero at 5%. The attenuation implies that much of the level fixed-effects pattern follows slowly changing city trajectories rather than tightly synchronized year-to-year movements. It supports a structural interpretation of persistence, but not a causal capability effect.

Additional controls do not erase the principal contrast. In the 2015–2019 specification including urbanization, trade openness, text-based environmental regulation intensity, and three further policy pilots, lagged digital infrastructure remains positive (beta = 0.00470, *p* = 0.0030), financial development remains negative (beta = −0.00254, *p* = 0.0192), and environmental digital governance remains positive (beta = 0.00143, *p* = 0.0332). More importantly, a common 2015–2018 sample holds both cities and years constant at 280 cities: digital financial inclusion remains negative (beta = −0.00780, *p* = 0.0099), environmental digital governance positive (beta = 0.00125, *p* = 0.0074), and digital infrastructure positive but imprecise (beta = 0.00190, *p* = 0.1515). The digital constructs therefore differ even when sample composition is fixed, although policy endogeneity remains possible.

### 4.4. The Scale–Composition Divide

Table 5 compares the associations of energy demand with absolute renewable consumption, total renewable share, and non-hydropower renewable share.

The scale–composition divide is the paper’s clearest empirical result. When the outcome is absolute renewable consumption, total energy use has a large positive contemporaneous coefficient (beta = 0.8696, *p* < 0.001) and a positive one-year-lagged coefficient (beta = 0.6015, *p* < 0.001). Energy intensity is also positively associated with absolute renewable consumption. Larger energy systems, unsurprisingly, consume more renewable energy in physical terms.

That conclusion reverses when transition is defined compositionally. Total energy use is negatively associated with total renewable share in the contemporaneous model (beta = −0.00733, *p* = 0.0308), while its lagged estimate is imprecise. Yet total energy use is positively associated with wind plus solar share both contemporaneously (beta = 0.00875, *p* < 0.001) and with a one-year lag (beta = 0.00792, *p* = 0.0024). The positive aggregate relationship thus combines growth in system scale with growth in non-hydropower renewables, without establishing broad substitution toward renewables. The chosen outcome determines which process is being described.

### 4.5. System State Nonlinearity and Fragile Complementarities

Table 6 reports the entropy-state and capability-by-entropy estimates used to assess system-state nonlinearity and complementarity.

H1 receives the strongest support. For total renewable share, full-mix Shannon entropy has a negative linear term (beta = −0.00341, *p* = 0.0170) and a positive squared term (beta = 0.00198, *p* < 0.001). Curvature persists with lagged full-mix entropy (squared beta = 0.00220, *p* < 0.001), normalized Simpson diversity (squared beta = 0.00169, *p* = 0.0011), and seven-carrier Shannon entropy excluding hydropower, wind, and solar (linear beta = −0.00866, *p* < 0.001; squared beta = 0.00152, *p* = 0.0010). Alternative specifications for non-hydropower share also retain significant entropy associations. The nonlinear pattern is therefore not solely mechanical overlap between renewable shares and the full-mix entropy index, although its exact shape remains outcome and index dependent.

The robustness comparison serves different purposes for H1 and H3. For H1, persistence across indices asks whether the nonlinear system state association survives alternative measures of portfolio diversity. For H3, the renewable-excluded index applies a stricter test: full-mix entropy contains the renewable carriers also used to construct the outcome, so its interactions may partly capture shared compositional information; removing hydropower, wind, and solar asks whether capability effects still vary with diversity in the remaining non-renewable portfolio.

The stronger complementarity claim does not survive this stricter specification. With full-mix entropy, interactions for digital infrastructure, industrial advancement, and financial development are positive for total renewable share after false discovery rate adjustment, and two interactions remain for non-hydropower share. Once renewable carriers are removed from entropy, however, none of the ten capability interactions survives adjustment (all adjusted *p* values >= 0.571); the bundle by entropy term is also imprecise (beta = −0.00137, *p* = 0.320). The loss of significance indicates that the apparent complementarity is specific to the full energy-mix configuration rather than a stable interaction with background portfolio diversity. H3 is therefore not supported as a general proposition. The full-mix interactions identify exploratory configuration patterns, not robust capability system complementarities.

**Figure 3:**
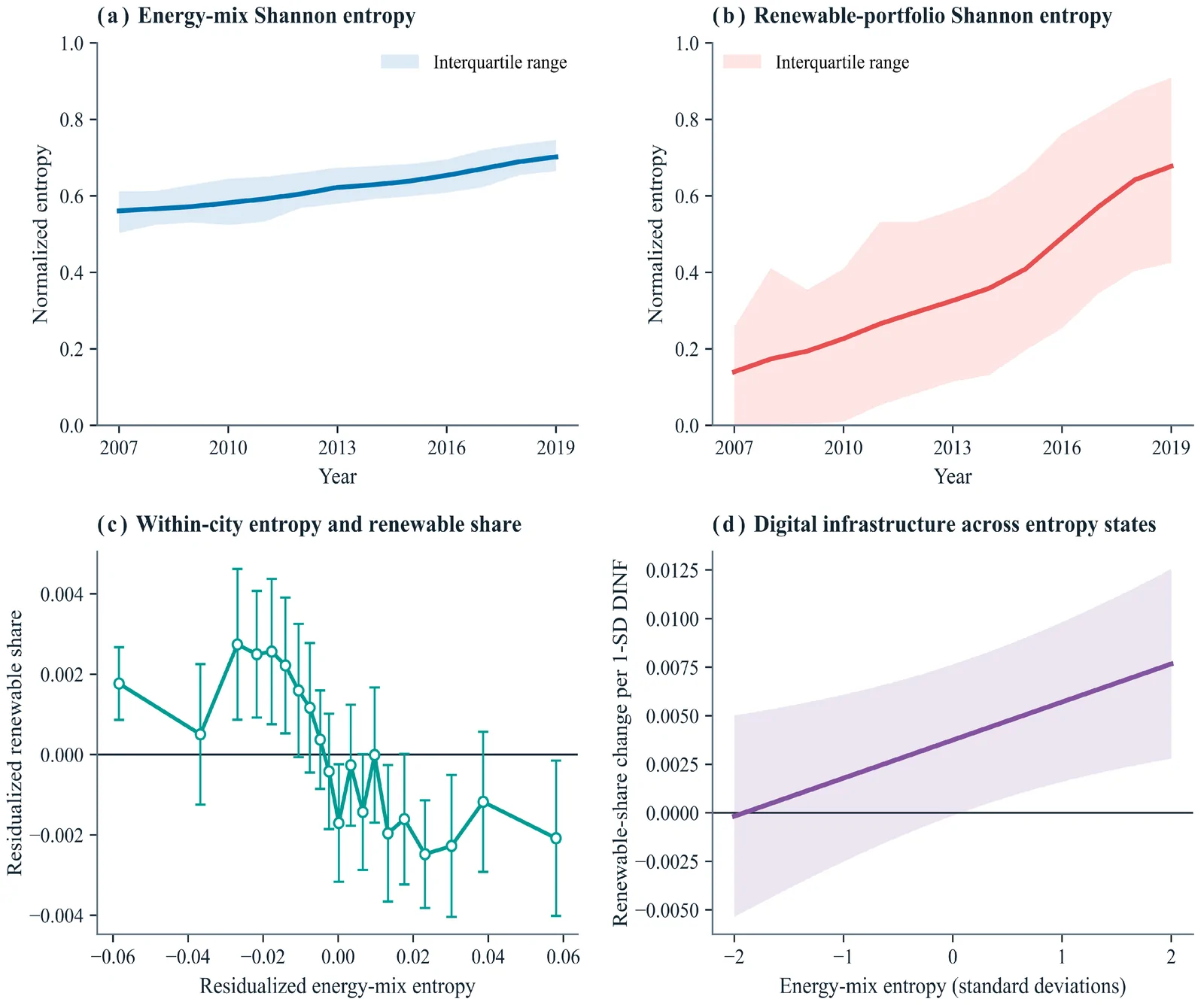
Energy system entropy and renewable transition outcomes. Panels show annual distributions, residualized binned patterns, and the digital infrastructure association across entropy states.

Figure 3 visualizes energy-portfolio entropy and its associations with renewable-transition outcomes. Entropy changes gradually over time but retains substantial cross-city dispersion; the residualized bins show the nonlinear composition pattern, and the marginal association panel shows how the digital infrastructure coefficient varies across observed entropy states. Because entropy and renewable shares are both functions of energy composition, the panels map conditional system configurations. They do not show that an intervention that raises entropy would itself change renewable share.

### 4.6. Mechanism-Consistent Patterns and Capability Bundles

Table 7 reports the channel-consistent associations, human-capital moderation estimates, and capability-bundle specifications.

The proposed innovation channel receives suggestive, not causal, support. Lagged digital infrastructure is positively associated with subsequent green innovation at the 10% level (beta = 0.0812, *p* = 0.0803). When lagged digital infrastructure and green innovation enter the renewable share model together, green innovation remains positive (beta = 0.00280, *p* = 0.0258) while the infrastructure coefficient becomes smaller and imprecise. This coefficient pattern is consistent with infrastructure operating partly through innovation, but causal mediation would require substantially stronger sequential exogeneity assumptions.

Other tests work against a simple complementarity story. The digital infrastructure by human capital interaction is negative (beta = −0.00215, *p* = 0.0105). With full-mix entropy, the equal-weight capability bundle has a null linear term, a negative squared term (beta = −0.00402, *p* = 0.0012), and a positive bundle by entropy interaction (beta = 0.00356, *p* = 0.0026); that interaction becomes imprecise when entropy excludes renewable carriers. The bundle is not a validated latent construct, and these results reject additive accumulation without identifying a stable alternative mechanism.

### 4.7. Distributional Heterogeneity as a Boundary Test

**Figure 4:**
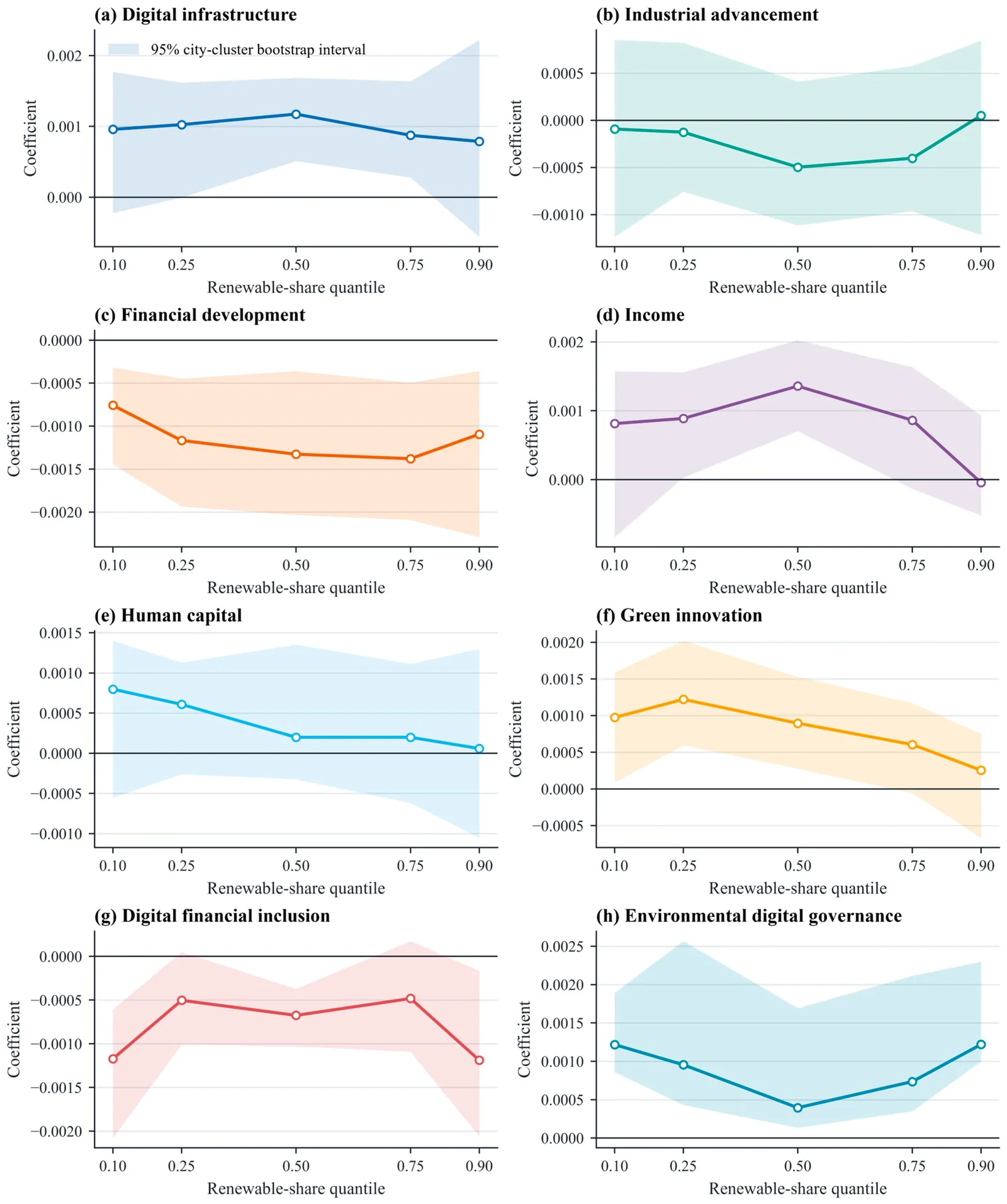
Adjusted quantile coefficient paths for renewable share. Outcomes and predictors are residualized for controls and city and year fixed effects. Shading gives 95% intervals from 99 city clustered bootstrap replications conditional on residualization.

The adjusted coefficient paths are heterogeneous in appearance but weak in formal comparison. Digital infrastructure and green innovation are generally positive, financial development and digital financial inclusion are negative, and environmental digital governance is positive but nonmonotonic. None of the eight tau = 0.90 minus tau = 0.10 contrasts survives false discovery rate adjustment; the unadjusted green innovation contrast (*p* = 0.020), for example, has an adjusted *p* value of 0.162. H2 is therefore not supported. Figure 4 describes conditional profiles, but it does not establish that capability associations systematically strengthen among high renewable share cities.

**Figure 5:**
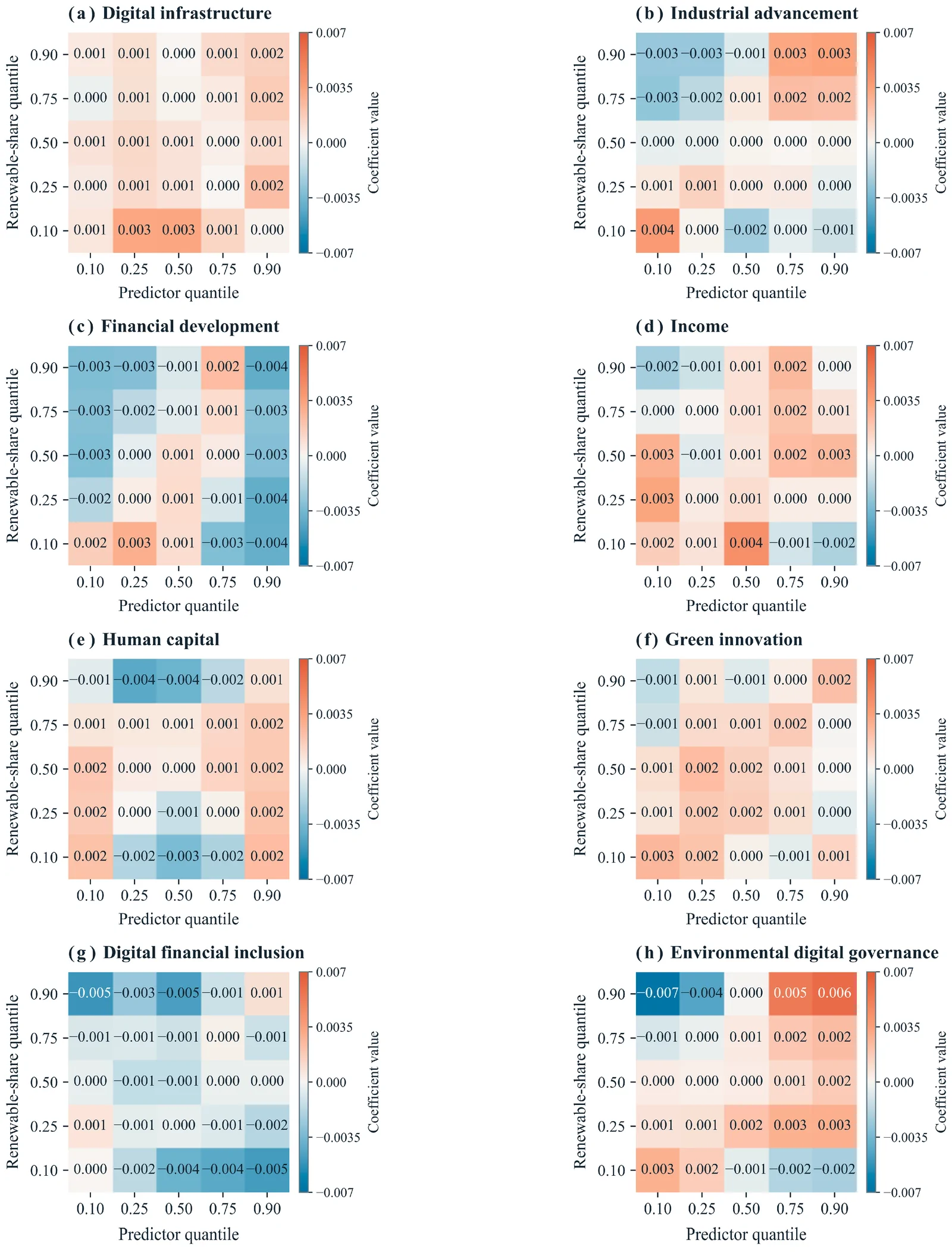
Adjusted local quantile coefficient surfaces. Each panel has its own coefficient value colorbar. The surfaces are descriptive maps and are not interpreted as independent significance tests.

Figure 5 introduces predictor state variation into this descriptive map. Several surfaces change sign across combinations of outcome and predictor quantiles, illustrating how a single average fixed effects coefficient can conceal localized patterns. The 25 cells in each panel are not independent significance tests, and the surfaces supply no separate causal identification. Their contribution is diagnostic: they locate the parts of the joint conditional distribution that generate apparent heterogeneity, with full numerical values reported in the Supplementary Table S2.

City examples further show why quantiles are not development stages. In 2019, Suihua lies near the 10th percentile of renewable share, Kaifeng near the median, and Chengdu near the 90th percentile. These are city-year positions, not permanent classifications. Chengdu combines a high total renewable share with a relatively small non-hydropower share, demonstrating how hydropower and resource geography can place a city in the upper tail without implying stronger governance capacity.

### 4.8. Structural Heterogeneity as a Boundary Test

**Figure 6:**
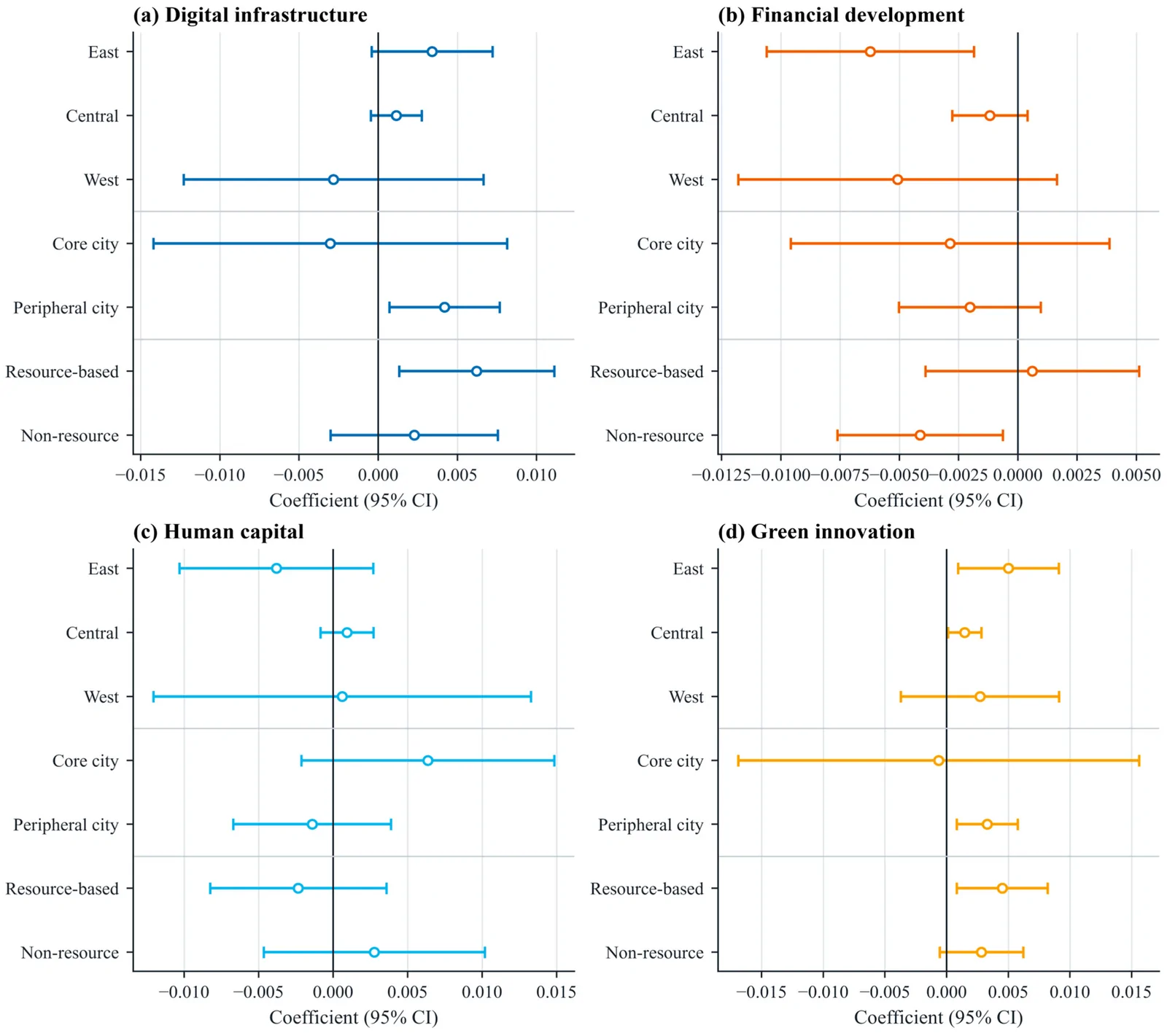
Regional, city hierarchy, and resource status estimates. Predictors use common full-sample standardization; points show one-year-lagged fixed effect coefficients and bars show 95% city-clustered confidence intervals.

Figure 6 and Table 8 report group-specific estimates across regions, city hierarchy, and resource status.

Several within-group estimates are precise: digital infrastructure is positive in peripheral and resource-based cities; financial development is negative in eastern and non-resource cities; and green innovation is positive in eastern, central, peripheral, and resource-based cities. Direct equality tests, however, provide the relevant evidence for between-group differences. No regional omnibus test, city hierarchy contrast, or resource status contrast survives false discovery rate adjustment (minimum adjusted *p* = 0.540). H4 is therefore not supported.

The subgroup estimates remain useful as descriptive checks on a single cumulative capability narrative, but they cannot distinguish agglomeration, policy sorting, incumbent industry exposure, or resource endowment as causal mechanisms. Nor does significance in one group and insignificance in another establish unequal coefficients. The policy discussion therefore uses observable energy profiles rather than inferred structural rankings.

## 5. Discussion and Policy Implications

### 5.1. What the Evidence Establishes

The evidence changes how urban renewable progress should be read. Absolute renewable consumption answers how much renewable energy a city uses; total renewable share asks whether renewables have gained relative weight; and non-hydropower share identifies the position of wind and solar. The positive energy use coefficient in the absolute model and the negative coefficient in the total share model show that renewable expansion can occur without equivalent compositional substitution. Transition assessments based on volume alone can therefore overstate structural change.

The digital results deliver a parallel measurement lesson. Broadband access, digital financial inclusion, and environmental digital governance remain empirically distinct in the identical 2015–2018 city-year sample: digital finance is negative, environmental digital governance is positive, and broadband infrastructure is imprecisely estimated in the joint model. These conditional associations do not rank governance quality. They show that infrastructure, financial access, and administrative procurement should enter theory and policy as separate capacities.

The complexity contribution is both substantive and bounded. Nonlinear system state associations persist when entropy is lagged, replaced by Simpson diversity, or calculated without renewable carriers. Capability-by-entropy interactions, in contrast, depend on the entropy construction and disappear when hydropower, wind, and solar are removed. The supported proposition is therefore that renewable composition is nonlinearly associated with portfolio configuration. Stable complementarity among urban subsystems remains an open hypothesis.

The structural evidence imposes a second boundary. Positive capability estimates do not occur only in core or eastern cities, so the descriptive pattern is not a simple agglomeration gradient. Yet formal coefficient difference tests do not confirm regional, hierarchical, or resource-based heterogeneity after multiple testing adjustment. Agglomeration, resource endowment, policy sorting, and intercity spillovers remain rival explanations rather than mechanisms eliminated by the design.

### 5.2. Why Finance, Skills, and Innovation Diverge

Financial depth is not the same as transition finance. Deposits plus loans relative to GDP do not reveal whether credit supports renewable generation, grid flexibility, incumbent industry, real estate, or consumption. The negative main coefficient therefore identifies an allocation problem, not an intrinsic cost of finance. Likewise, the positive finance interaction with full-mix entropy is too index sensitive to establish that diversification changes finance’s causal effect.

The human capital result reflects a similar gap between a broad proxy and a specific mechanism. Higher education enrollment measures general learning capacity, not electrical engineering, installation, grid operation, digital control, or transition management. Research on green skills and knowledge spillovers emphasizes task match and absorptive capacity [21,24]. A null main effect and a negative broadband interaction therefore do not show that skills are irrelevant; they show that enrollment alone cannot identify workforce readiness for energy transition.

Green innovation has the most consistent positive capability association. Its contemporaneous and lagged coefficients, together with the infrastructure channel specification, are consistent with technological accumulation. The first-difference coefficient is nevertheless null, and the entropy interaction does not survive multiple testing adjustment. Patents may precede deployment by several years, diffuse across cities, or fail to be commercialized locally. The evidence supports an innovation-consistent pathway but not causal mediation.

### 5.3. Policy by Observable Transition State

Policy should begin with a diagnosis of the energy portfolio rather than a fixed city label. A city with low total and non-hydropower shares faces a different constraint from one with a high total share dominated by hydropower. The former may lack project pipelines, grid access, or procurement capacity; the latter may need diversification, storage, and transmission coordination. Cities with rising wind and solar shares face yet another problem: forecasting, flexible demand, and balancing become more valuable as variable generation expands. Table 9 summarizes the corresponding policy diagnostics based on observable transition outcomes.

The 2019 city positions illustrate this diagnostic logic. Suihua is near the 10th percentile of renewable share, Kaifeng near the median, and Chengdu near the 90th percentile. These examples are not treatment groups or permanent rankings; they indicate where a policymaker can begin inspecting the underlying portfolio. Chengdu’s upper-tail position partly reflects hydropower, so a target based only on total renewable share could overlook the separate need for wind and solar diversification.

For low-share cities, the immediate priorities are reliable broadband access, transparent project information, grid readiness assessment, and procurement capacity. In more diversified systems, digital infrastructure should be tied to metering, dispatch, and demand response rather than treated as a generic technology target. Descriptive estimates for resource-based and peripheral cities can help identify where closer diagnosis is warranted, but the nonsignificant coefficient difference tests do not justify assuming that these groups benefit more.

Financial policy should be technology- and use-specific. Green bonds, transition finance standards, credit guarantees, and disclosure requirements can direct capital toward grid flexibility, storage, and renewable projects. Expanding deposits and lending without sectoral safeguards is not equivalent to transition finance. Cities with established project portfolios may emphasize verification and risk pricing; cities with thin pipelines may first require standardized projects and credit enhancement.

Workforce policy should likewise move from enrollment targets to skills mapping. Cities can identify shortages in grid operation, power electronics, project finance, data engineering, and environmental regulation, then align vocational and university programs with local deployment plans. This approach responds directly to the weak performance of the broad human capital proxy and to evidence that green transition readiness depends on task specific skills [21].

Portfolio policy must also account for distribution and resilience. Renewable expansion can distribute costs and benefits unevenly, while rapid infrastructure change can create supply chain and land-use risks [35,36,37]. Composition targets should therefore be evaluated alongside affordability, employment transition, system reliability, and local participation.

### 5.4. Limitations and Interpretation Boundary

The conclusions are bounded in six ways. First, the observational fixed-effects design does not establish causality; reverse causality and time-varying policy endogeneity remain possible. Second, city-level energy data aggregate users and districts. Third, total renewable consumption combines hydro, wind, and solar, although the non-hydropower outcome separates the latter two. Fourth, full-mix entropy shares components with the renewable outcomes; alternative entropy measures reduce this concern for H1 but expose the fragility of H3. Fifth, the quantile bootstrap is conditional on residualization, and the local surfaces are descriptive. Sixth, intercity spillovers are not estimated because no prespecified spatial weights matrix is available. These limits rule out causal and universal interpretations, but they do not alter the documented scale–composition divide or the robust nonlinear entropy association.

## 6. Conclusions

Urban renewable progress has two dimensions that need not move together: the volume of renewable energy and its share of the energy mix. Across 284 Chinese cities, total energy use is strongly positively associated with absolute renewable consumption, negatively associated with total renewable share, and positively associated with wind and solar share. More renewable energy can therefore coexist with incomplete compositional transition.

Energy portfolio state helps explain this divergence without supplying a universal development ladder. Renewable composition has a nonlinear association with entropy that persists across temporal ordering and alternative diversity measures. In contrast, capability complementarities, upper–lower quantile differences, and structural coefficient differences are not robust to alternative entropy construction or multiple testing adjustment. The evidence supports system state nonlinearity while rejecting stronger claims of uniform capability accumulation.

The capability results reinforce that boundary. Digital infrastructure, digital financial inclusion, and environmental digital governance are empirically distinct; broad financial depth, general human capital enrollment, and green innovation also capture different mechanisms. Their coefficients should not be converted into a single ranking of city readiness or into causal policy effects.

The practical implication is diagnostic. Policymakers should first determine whether a city faces a scale problem, a portfolio composition problem, a coordination problem, or a capability allocation problem, and only then match broadband, finance, skills, innovation, and grid policy to that constraint. Measuring the portfolio before prescribing the capability is the central lesson of the analysis.

## Figures and Tables

**Table 1:** Variable definitions and coverage.

Code	Construct	Measurement	Unit	Source and Years
RED	Renewable share	(hydro + wind + solar)/total final energy	Proportion	Energy dataset [3], 2007–2019
RED-NH	Non-hydro renewable share	(wind + solar)/total final energy	Proportion	Energy dataset [3], 2007–2019
RED-A	Absolute renewable use	Ln (1 + hydro + wind + solar)	Log of 10,000 tce	Energy dataset [3], 2007–2019
EC	Total final energy use	Sum of ten carriers; level in Equation (1), ln(EC) in regressions	10,000 tce; log in models	Energy dataset [3], 2007–2019
ECI	Energy intensity	Ln (total final energy/city GDP)	Log ratio	Energy data + [32], 2007–2019
DINF	Digital infrastructure	Ln (1 + broadband subscriptions per 100 registered residents)	Log rate	Public city panel [32], 2007–2019
DFI	Digital financial inclusion	Peking University DFI Index	Index	PKU DFIIC [31], 2011–2019
EDG	Environmental digital governance	Cumulative environment-related digital procurement/GDP	Ratio	Mendeley Data [33], 2015–2019
GTI	Green innovation	Ln (1 + green invention and utility model patent applications)	Log count	Dryad [34], 2007–2018
AIS	Industrial advancement	Tertiary industry value added/secondary industry value added	Ratio	Public city panel [32], 2007–2019
FD	Financial development	Year-end bank deposits plus loans/GDP	Ratio	Public city panel [32], 2007–2019
GDP	Income	Ln (city GDP per capita in yuan)	Log yuan	Public city panel [32], 2007–2019
HCI	Human capital	Regular higher education enrollment/registered population	Percent	Public city panel [32], 2007–2019
H10	Energy-mix entropy	Normalized Shannon entropy of ten carrier shares	0–1 index	Constructed from [3], 2007–2019
H3	Renewable entropy	Normalized Shannon entropy of hydro, wind, and solar shares	0–1 index	Constructed from [3], 2007–2019

Note: The core balanced sample contains 3692 observations and 284 cities. Continuous predictors are standardized within each estimation sample. Energy quantities are measured in 10,000 tons of standard coal equivalent (tce) before the transformations shown. Supplementary Table S8 reports model-specific coverage.

**Table 2:** Descriptive statistics.

Variable	N	Mean	SD	Min	Median	Max
RED	3692	0.0522	0.0458	0.0003	0.0388	0.2526
DINF	3692	2.6202	0.7891	0.0000	2.6515	5.2516
ECI	3692	−0.5947	0.4532	−1.8537	−0.6237	1.4679
GTI	3396	4.2473	1.7941	0.0000	4.1666	9.9617
AIS	3692	0.9231	0.5106	0.1087	0.8068	5.1610
FD	3692	2.2310	1.0474	0.5600	1.9287	7.1794
GDP	3692	10.4795	0.6705	7.6660	10.4923	12.3237
HCI	3692	1.6246	1.9248	0.0268	0.9326	12.7643
DFI	2556	165.5389	65.4176	17.0200	171.9301	321.6457
EDG	1400	0.0001	0.0004	0.0000	0.0000	0.0037
RED NH	3692	0.0081	0.0117	0.0000	0.0035	0.0854
H10	3692	0.6219	0.0853	0.3682	0.6285	0.8395
H3	3692	0.3670	0.3025	−0.0000	0.3127	0.9800

Note: Variable codes and units are defined in Table 1. Coverage differs for GTI, DFI, and EDG.

**Table 3:** Baseline city and year fixed-effect estimates for renewable share.

Predictor	Separate FE Beta (SE)	*p*	Joint Core FE Beta (SE)	*p*	N
Digital infrastructure	0.0033 (0.0021)	0.110	0.0039 ** (0.0019)	0.037	3692
Industrial advancement	−0.0009 (0.0011)	0.444	−0.0003 (0.0011)	0.815	3692
Financial development	−0.0044 *** (0.0016)	0.006	−0.0048 *** (0.0017)	0.004	3692
Income	0.0044 ** (0.0021)	0.039	−0.0011 (0.0024)	0.643	3692
Human capital	0.0022 (0.0029)	0.436	0.0030 (0.0029)	0.307	3692
Green innovation	0.0036 *** (0.0013)	0.006	−	−	3396
Digital financial inclusion	−0.0143 *** (0.0044)	0.001	−	−	2556
Environmental digital governance	0.0019 *** (0.0007)	0.007	−	−	1400

Note: All models include city and year fixed effects, logged registered population, and four policy pilot controls. Standard errors clustered by city are in parentheses. Predictors are standardized. *** *p* < 0.01; ** *p* < 0.05.

**Table 4:** One-year-lag and first-difference estimates.

Predictor	Lagged FE Beta (SE)	*p*	First-Difference Beta (SE)	*p*	N (lag/diff)
Digital infrastructure	0.0033 (0.0021)	0.112	−0.0002 (0.0002)	0.395	3408/3408
Industrial advancement	−0.0009 (0.0013)	0.496	−0.0002 (0.0002)	0.125	3408/3408
Financial development	−0.0023 * (0.0014)	0.099	−0.0002 * (0.0001)	0.095	3408/3408
Income	0.0032 (0.0022)	0.153	−0.0001 (0.0001)	0.272	3408/3408
Human capital	0.0017 (0.0031)	0.583	6.87 × 10^−5^ (0.0002)	0.657	3408/3408
Green innovation	0.0033 *** (0.0013)	0.009	0.0002 (0.0002)	0.448	3396/3113
Digital financial inclusion	−0.0107 *** (0.0033)	0.001	−0.0009 ** (0.0004)	0.033	2272/2272
Environmental digital governance	0.0014 ** (0.0007)	0.036	0.0007 *** (0.0002)	0.003	1120/1120

Note: Lagged models include city and year fixed effects; first-difference models include year fixed effects. Both use the baseline controls and city-clustered standard errors. Standard errors clustered by city are in parentheses. Predictors are standardized. *** *p* < 0.01; ** *p* < 0.05; * *p* < 0.10.

**Table 5:** Energy demand: scale and composition outcomes.

Outcome	Energy Measure	Timing	Beta (SE)	*p*	N
Absolute renewable use	Total energy use	Current	0.8696 *** (0.0808)	4.97 × 10^−27^	3692
Absolute renewable use	Total energy use	Lag 1	0.6015 *** (0.0979)	7.93 × 10^−10^	3408
Absolute renewable use	Energy intensity	Current	0.3853 *** (0.0445)	4.70 × 10^−18^	3692
Absolute renewable use	Energy intensity	Lag 1	0.2641 *** (0.0516)	3.14 × 10^−7^	3408
Renewable share	Total energy use	Current	−0.0073 ** (0.0034)	0.031	3692
Renewable share	Total energy use	Lag 1	−0.0038 (0.0034)	0.262	3408
Non hydro share	Total energy use	Current	0.0088 *** (0.0023)	0.000	3692
Non hydro share	Total energy use	Lag 1	0.0079 *** (0.0026)	0.002	3408

Note: All models include the baseline controls and city and year fixed effects. Continuous energy measures are standardized. Standard errors clustered by city are in parentheses. Predictors are standardized. *** *p* < 0.01; ** *p* < 0.05.

**Table 6:** Entropy state and capability-by-entropy estimates.

Outcome	Entropy	Term	Beta (SE)	*p*	FDR *p*
Renewable share	Full energy mix	Entropy	−0.0034 ** (0.0014)	0.017	-
Renewable share	Full energy mix	Entropy squared	0.0020 *** (0.0005)	1.43 × 10^−5^	-
Renewable share	Renewable portfolio	Entropy	−0.0018 ** (0.0008)	0.021	-
Renewable share	Renewable portfolio	Entropy squared	−0.0021 *** (0.0006)	0.000	-
Non hydro share	Full energy mix	Entropy	−0.0014 ** (0.0007)	0.037	-
Non hydro share	Full energy mix	Entropy squared	0.0005 * (0.0003)	0.078	-
Non hydro share	Renewable portfolio	Entropy	0.0020 *** (0.0006)	0.001	-
Non hydro share	Renewable portfolio	Entropy squared	0.0032 *** (0.0003)	1.40 × 10^−35^	-
Renewable share	Full energy mix	Digital infrastructure × entropy	0.0020 ** (0.0008)	0.016	0.032
Renewable share	Full energy mix	Industrial advancement × entropy	0.0017 ** (0.0007)	0.016	0.032
Renewable share	Full energy mix	Financial development × entropy	0.0025 *** (0.0009)	0.005	0.019
Renewable share	Full energy mix	Human capital × entropy	−0.0014 * (0.0007)	0.053	0.088
Renewable share	Full energy mix	Green innovation × entropy	0.0011 (0.0008)	0.161	0.179
Non hydro share	Full energy mix	Digital infrastructure × entropy	0.0006 (0.0004)	0.122	0.153
Non hydro share	Full energy mix	Industrial advancement × entropy	0.0015 *** (0.0005)	0.004	0.019
Non hydro share	Full energy mix	Financial development × entropy	0.0017 *** (0.0006)	0.006	0.019
Non hydro share	Full energy mix	Human capital × entropy	−5.91 × 10^−6^ (0.0004)	0.989	0.989
Non hydro share	Full energy mix	Green innovation × entropy	−0.0005 (0.0003)	0.114	0.153

Note: City and year fixed effects with city-clustered standard errors. Interaction family *p* values use the Benjamini–Hochberg false discovery rate adjustment. Standard errors clustered by city are in parentheses. Predictors are standardized. *** *p* < 0.01; ** *p* < 0.05; * *p* < 0.10.

**Table 7:** Channel consistent, moderation, and capability bundle estimates.

Model	Term	Beta (SE)	*p*	N
DINF > GTI	Lagged DINF	0.0812 * (0.0464)	0.080	3113
RED without GTI	Lagged DINF	0.0024 (0.0019)	0.196	3113
RED with GTI	Lagged DINF	0.0023 (0.0018)	0.221	3113
RED with GTI	GTI	0.0028 ** (0.0013)	0.026	3113
DINF HCI moderation	Lagged DINF	0.0030 (0.0019)	0.116	3408
DINF HCI moderation	HCI	0.0048 (0.0030)	0.112	3408
DINF HCI moderation	Lagged DINF × HCI	−0.0021 ** (0.0008)	0.011	3408
Capability bundle	Capability bundle	−0.0004 (0.0027)	0.895	3396
Capability bundle	Capability bundle squared	−0.0040 *** (0.0012)	0.001	3396
Capability bundle	Energy-mix entropy	−0.0034 ** (0.0015)	0.024	3396
Capability bundle	Bundle × entropy	0.0036 *** (0.0012)	0.003	3396

Note: These patterns are described as channel-consistent associations; they are not causal mediation estimates. Standard errors clustered by city are in parentheses. Predictors are standardized. *** *p* < 0.01; ** *p* < 0.05; * *p* < 0.10.

**Table 8:** Lagged capability estimates by region, city hierarchy, and resource status.

Subgroup	DINF Beta (SE)	FD Beta (SE)	HCI Beta (SE)	GTI Beta (SE)
East	0.0034 * (0.0019)	−0.0062 *** (0.0022)	−0.0038 (0.0033)	0.0050 ** (0.0021)
Central	0.0011 (0.0008)	−0.0012 (0.0008)	0.0009 (0.0009)	0.0015 ** (0.0007)
West	−0.0028 (0.0048)	−0.0051 (0.0034)	0.0006 (0.0065)	0.0027 (0.0033)
Core city	−0.0030 (0.0057)	−0.0029 (0.0034)	0.0064 (0.0043)	−0.0006 (0.0083)
Peripheral city	0.0042 ** (0.0018)	−0.0020 (0.0015)	−0.0014 (0.0027)	0.0033 *** (0.0013)
Resource-based	0.0062 ** (0.0025)	0.0006 (0.0023)	−0.0023 (0.0030)	0.0045 ** (0.0019)
Non-resource	0.0023 (0.0027)	−0.0041 ** (0.0018)	0.0028 (0.0038)	0.0028 (0.0017)

Note: One-year-lagged predictors; common full-sample predictor standardization; baseline controls; city and year fixed effects; city-clustered standard errors. Subgroup observations and direct coefficient difference tests are reported in Supplementary Table S5. Standard errors clustered by city are in parentheses. Predictors are standardized. *** *p* < 0.01; ** *p* < 0.05; * *p* < 0.10.

**Table 9:** Policy diagnostic matrix based on observable transition outcomes.

Observable Profile	Principal Constraint	Priority Interventions
Low total renewable share; low wind plus solar share	Weak project pipeline, grid access, and administrative readiness	Resource assessment; transparent procurement; standardized project pipelines; broadband-enabled project information
High total renewable share; low wind plus solar share	Portfolio concentration, often with hydropower dependence	Wind and solar diversification; storage; transmission coordination; flexible dispatch
Rising wind plus solar share; diversified energy mix	System integration, forecasting, and balancing	Smart metering; demand response; grid balancing; storage; project-level carbon market verification
Resource-based city with incumbent industry exposure	Carbon lock-in, credit allocation, and workforce transition	Use specific transition finance; skill mapping; worker transition support; green public procurement

Note: Profiles are diagnostic categories, not permanent city rankings or causal treatment groups.

## Data Availability

The harmonized city-year analysis panel, variable definitions, sample-coverage information, analysis code, and supplementary results are provided in the Supplementary Materials. The original datasets are available from the public sources cited in References [3,31,32,33,34]. No primary data were collected for this study.

## Supplementary Materials

The following supporting information can be downloaded at: https://www.mdpi.com/article/10.3390/e28090971/s1.
